# Temporary chest closure and open chest management for severe chest trauma: a retrospective study of 10 cases at a single hospital

**DOI:** 10.1007/s11748-025-02200-8

**Published:** 2025-09-16

**Authors:** Hiroyuki Kayata, Akihiro Usui, Koki Terakawa, Koichi Inukai, Yu Hashimoto, Fumitaka Kato, Koji Amano, Nobutaka Mukai, Naoki Shinyama, Masanori Morita

**Affiliations:** 1 Department of Trauma and Critical Care Medicine, Sakai City Medical Center, 1-1-1 Ebaraji-cho, Nishi-ku, Sakai, Osaka 593-8304 Japan; 2 Division of General Thoracic Surgery, Sakai City Medical Center, 1-1-1 Ebaraji-cho, Nishi-ku, Sakai, Osaka 593-8304 Japan

**Keywords:** Damage-control surgery, Damage-control thoracic surgery, Chest trauma, Complications, Trauma management

## Abstract

**Objectives:**

Evidence to establish standardized damage control surgery for severe chest trauma is insufficient. Therefore, we aimed to evaluate the outcomes, complications, effectiveness, and safety of temporary chest closure and open chest management in our hospital.

**Methods:**

We retrospectively reviewed the backgrounds and perioperative outcomes of 10 patients who underwent open chest management with temporary chest closure for severe trauma at our hospital from January 2015 to June 2024 using their medical records.

**Results:**

The median patient age was 54 years, nine patients had blunt multiple trauma, and one patient had an isolated, penetrating chest injury. All patients had hemorrhagic shock upon arrival: the median chest Abbreviated Injury Scale score and Injury Severity Score were 4.5 and 30, respectively. The initial chest surgery was thoracotomy-based hemostasis for injuries of the chest wall, lungs, heart, and great vessels in six cases, and pulmonary resection for lung injury in four cases; all cases involved open chest management with temporary chest closure after intrapleural gauze packing. The median operative time and intraoperative bleeding was 72 min and 1710 mL, respectively. Seven of the 10 patients survived, with a median open chest management period of 2 days, with no postoperative empyema or wound infection.

**Conclusion:**

Open chest management with temporary chest closure for severe chest trauma is useful for the prompt completion of the initial chest surgery and initiation of treatment for concomitant injuries and resuscitation in the intensive care unit.

## Introduction

In certain cases, damage control surgery (DCS) must be adopted to save the patient. These include cases in which the patient presents with the “lethal triad” (hypothermia, acidosis, and coagulopathy), presents with severe circulatory failure upon arrival at the hospital or during initial treatment, or requires massive blood transfusion. DCS is defined as the initial control of hemorrhage and contamination, followed by physiological resuscitation in the intensive care unit (ICU) and delayed, planned definitive surgery [1]. Over the past three decades, the concept of DCS has been widely accepted, and several comprehensive reviews have been published to describe its evolution and benefits [2–4]. DCSs were first applied to penetrating abdominal injuries, which resulted in improved survival; the principles were subsequently applied to vascular surgery, general emergency surgery, and orthopedics [1–3, 5–9]. However, the application of DCSs in thoracic trauma was delayed owing to the infrequency of cases in which the patient requires emergency thoracotomy [10–13]. However, in recent years, a growing number of such cases have been reported in which DCSs were implemented as effective treatment strategies [14–19].

One of the tactics in damage control thoracic surgery (DCTS) is open chest management (OCM) with temporary chest closure (TCC). OCM is considered useful as it allows for prompt therapeutic intervention for concomitant injuries owing to the quick completion of the initial chest surgery; it is also used to prevent ventilatory disturbance and a decreased cardiac output due to increased intrapleural pressure normally caused by intrapleural gauze packing [20–22]. However, a few reports on OCM with TCC for chest trauma have been published [20–22], and the evidence regarding its indications and the methods to use is insufficient. Therefore, we report on the treatment outcomes, complications, effectiveness, and safety of OCM with TCC for cases of severe chest trauma encountered at our hospital.

## Subjects and methods

### Study design and data collection

For this single-center, retrospective case series, we analyzed patients’ medical records. In total, 44 cases of emergency surgery for chest trauma were performed at our hospital from January 2015 to June 2024, of which those that involved OCM with TCC were included in this study. Cases of resuscitative thoracotomy for cardiac arrest on arrival and deaths in the emergency room were excluded.

From the medical records, we obtained the following data: age, sex, comorbidities, vital signs on arrival, detailed mechanism of injury, isolated thoracic trauma versus multiple trauma, thoracic trauma severity according to the Abbreviated Injury Scale (AIS) score, overall trauma severity according to the Injury Severity Score (ISS), and details of the concomitant injuries. We assessed surgical parameters (surgical procedures for thoracic injury, time from arrival to surgery, operation time, intraoperative blood loss and blood transfusion, and details of therapeutic intervention for concomitant injury) and treatment outcomes [re-thoracotomy for hemostasis, extracorporeal membrane oxygenation (ECMO), tracheotomy, postoperative complications, mortality, duration of OCM, postoperative mechanical ventilation, postoperative ICU stay, and postoperative hospital stay].

This study was approved by the Ethics Committee of our hospital in accordance with the provisions of the Declaration of Helsinki (approval no.: 25-497). Anonymity was ensured in accordance with the Japanese Act on the Protection of Personal Information. The need for informed consent was waived by the Committee owing to the retrospective nature of the study.

### Open chest management

To date, no guidelines on the indications for OCM have been published. The indications for OCM in our hospital (Table 1) are based on physiological parameters and injury characteristics that reportedly influence the decision whether to consider DCTS [17, 23–25]. However, in practice, we do not apply OCM if only one of the criteria listed in Table 1 is met. Rather, OCM is performed on patients who require thoracotomy in the setting of severe circulatory failure, those with the lethal triad, and those who require intrapleural hemostasis with gauze packing and prompt intervention for concomitant injuries. In particular, as physiological parameters change rapidly during the initial treatment, changes during the initial treatment should be considered rather than only the values at the time of admission. The final decision of whether to apply OCM is made by the surgeon, taking into account any concomitant organ injuries and the findings during surgery for chest trauma.

**Table 1:** Indications of damage control thoracic surgery, temporary chest closure, and open chest management at our hospital

Physiological parameter	Injury characteristic
Acidosis (pH < 7.2) Hypothermia (< 34 °C) Coagulopathy Hemodynamic instability Massive transfusion	Resuscitative thoracotomy with aortic occlusion The need to pack gauze over the thoracic wall and/or the chest cavity to control coagulopathic bleeding Development of thoracic compartment syndrome during an attempt at chest closure Concomitant nonthoracic injuries requiring intervention

**Fig. 1:**
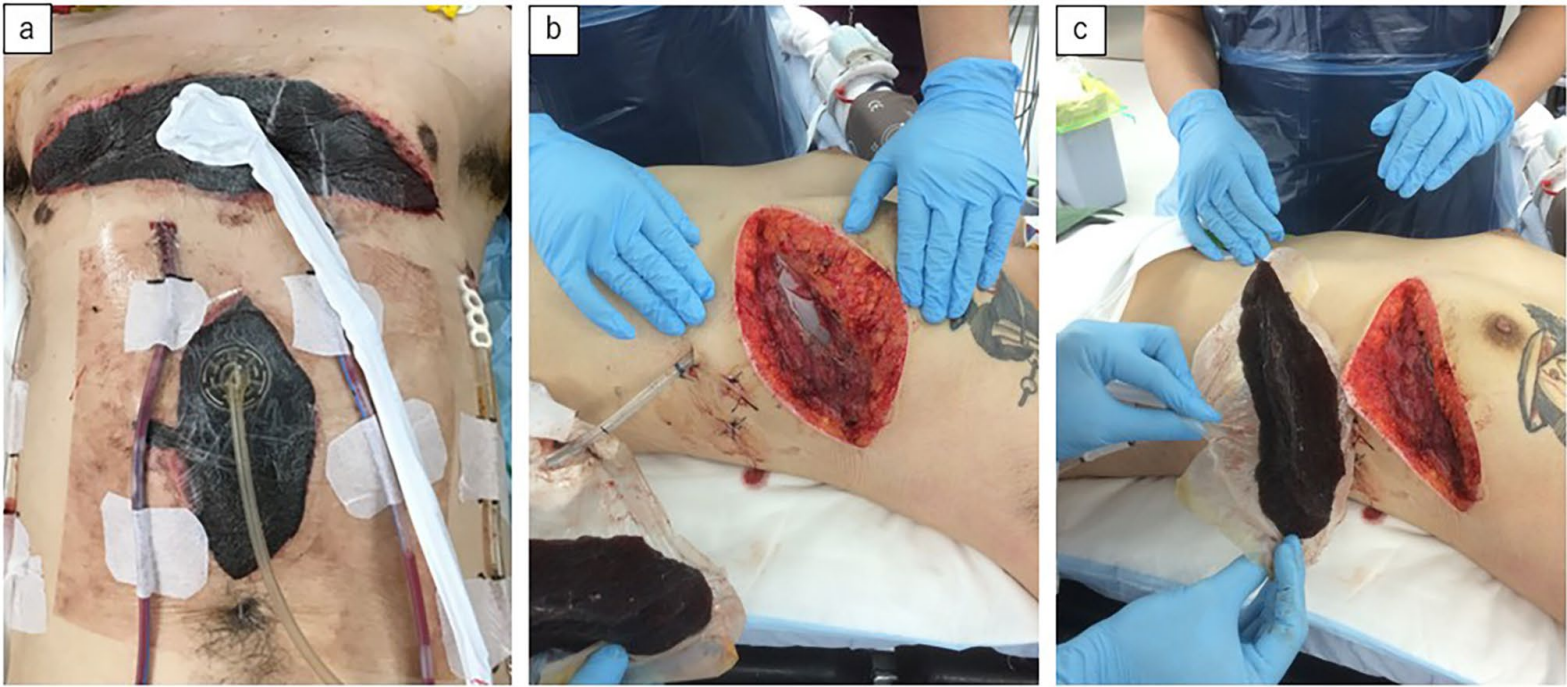
Examples of temporary chest closure and open chest management at our hospital. **a** A case in which thoracotomy and laparotomy were performed simultaneously. The patient’s abdomen was treated using a device for open abdominal management, and the chest was treated using a negative-pressure wound therapy device. **b**, **c** When the negative-pressure wound therapy device is used, SI Mesh (SI Mesh™ is a silicone gel dressing for wounds; it has a mesh structure) is used as a wound contact layer to protect the lung parenchyma. Subsequently, a foam dressing is used with the negative-pressure wound therapy device

After initial chest surgery, a chest tube is inserted and OCM is performed with TCC. At our hospital, TCC methods include closing only the skin or using adhesive dressing, negative-pressure wound therapy devices, or a commercial device for open abdominal management (Fig. 1). After the initial surgery, we perform planned reoperations every 24 to 48 h, and once hemostasis is achieved in the chest cavity, we promptly perform definitive chest closure, striving to minimize the OCM time. The chest tube is used to apply a pressure of − 10 to − 20 cmH_2_O, whereas the negative-pressure wound therapy device or the medical device for open abdominal management is used to apply a pressure of − 40 to − 80 mmHg.

Negative pressure wound therapy devices or medical devices for open abdominal management are used off-label for TCC and OCM; therefore, we obtain permission to use these devices via ethics review for each case, in accordance with hospital standards.

### Statistical analyses

Continuous variables are presented as medians [quartiles 1–3], and categorical data as n (%). Analyses were performed using IBM SPSS Statistics version 26 (IBM Corporation, Armonk, NY, USA).

## Results

The characteristics of the 10 included patients are listed in Table 2. Their median age was 54 years; only one patient had chest trauma alone (a penetrating), whereas the other nine patients had multiple blunt injuries. Their vital signs at presentation indicated circulatory failure, and the median lactate concentration upon arrival at the hospital was high (4.5 mmol/L). The median chest AIS score and ISS were 4.5 and 30, respectively. Most of the patients had severe multiple trauma with various concomitant injuries, including injuries of the head, abdominal organs and vessels, pelvis, spine, and soft tissues. Details of the initial chest surgery and subsequent interventions for concomitant injuries are summarized in Table 3. All patients were operated via open chest surgery in the supine position and underwent TCC and OCM with intrapleural gauze packing. The TCC methods were adhesive dressing in one case, negative-pressure wound therapy devices in eight cases, and a commercial device for open abdominal management in one case. The median operative time and intraoperative bleeding were 72 min and 1710 mL, respectively. After the initial chest surgery, one patient underwent laparotomy and five underwent interventional radiology (IVR) for hemostasis as interventions for concomitant injuries. Postoperative results are summarized in Table 4. The median duration of postoperative ventilation, ICU stay, and hospital stay were 8, 8, and 17 days, respectively, and the median duration of OCM was 2 days. Veno-veno and veno-arterial ECMO were required for postoperative respiratory and circulatory maintenance in three cases and one case, respectively, and tracheostomy was performed for prolonged respiratory failure in four cases. No infectious complications of the surgical site, such as wound infection or empyema, were observed.

**Table 2:** Clinical characteristics of 10 patients treated via open chest management

Parameter	Value
Age, years	54 [28–73]
Sex, male	6 (60)
Mechanism of injury	
Penetration	1 (10)
Blunt	9 (90)
Traffic accident	5 (50)
Fall	3 (30)
Run over by a train	1 (10)
Chest trauma alone	1 (10)
Multiple trauma	9 (90)
Vital signs on arrival	
SBP, mmHg	80 [78–119]
HR, beats per min	118 [100–126]
Shock index	0.87 [0.64–1.03]
SpO_2_, 10–15 L oxygen administration	98 [94–99]
RR, breaths per min	26 [21–30]
Lactate on arrival (mmol/L)	4.5 [3.3–10.9]
Chest AIS score	4.5 [4–5]
ISS	30 [22–40]
Details of chest injury	
Lung injury	6 (60)
Heart or great vessels	1 (10)
Thoracic wall	7 (70)
Details of concomitant injuries	
Head	1 (10)
Abdominal	4 (40)
Pelvis, spine, or extremities	4 (40)
Great vessels	1 (10)
Soft tissue	1 (10)

Some of the patients had more than one concomitant injury

Continuous data are shown as medians [quartiles 1–3]. Categorical data are shown as *n* (%)

*AIS* Abbreviated Injury Scale, *HR* heart rate, *ISS* Injury Severity Score, *RR* respiratory rate, *SBP* systolic blood pressure, *SBP/HR* shock index, *SpO*_*2*_ blood oxygen saturation

**Table 3:** Details of thoracic surgery and subsequent interventions for concomitant injuries in 10 patients

Parameters	Values
Surgical procedure for chest trauma	
Thoracotomy for hemostasis	6 (60)
Lung resection	4 (40)
Suturing of cardiac injury	1 (10)
Repair of aorta	1 (10)
Intrathoracic gauze packing	10 (10)
Method of temporary chest closure	
Skin closure only	1 (10)
Using a negative-pressure wound therapy device	9 (90)
Surgical time (min)	72 [50–97]
Intraoperative bleeding (mL)	1710 [1225–2225]
Intraoperative blood transfusion (units)	
RBCs	13 [8–18]
FFP	12 [8–12]
PLTs	5 [0–17.5]
Additional procedure for concomitant injuries	
Laparotomy for hemostasis	1 (10)
Interventional radiology	5 (50)

Some the patients underwent more than one surgical procedure for chest trauma

Continuous data are shown as medians [quartiles 1–3]. Categorical data are shown as *n* (%)

*FFP* fresh-frozen plasma, *RBC* red blood cell, *PLT* platelet

**Table 4:** Details of postoperative outcomes

Parameter	Value
In-hospital mortality	3 (30)
Postoperative length of ventilation, days	8 [3–33]
Postoperative length of ICU stay, days	8 [6–26]
Postoperative length of hospital stay, days	17 [8–52]
Open chest management, days	2 [2–4]
Postoperative complications	
Emergency re-operation for chest hemostasis^a^	2 (20)
Pneumonia	3 (30)
Surgical site infection	0 (0)
Empyema	0 (0)
V-V ECMO	3 (30)
V-A ECMO	1 (10)
Tracheostomy	4 (40)

Continuous data are shown as median [quartiles 1–3]. Categorical data are shown as *n* (%)

*ICU* intensive care unit, *V-A ECMO* veno-arterial extracorporeal membrane oxygenation, *V-V ECMO* veno-veno extracorporeal membrane oxygenation

^a^Emergency re-operation in the ICU within 12 h of admission

## Discussion

In our hospital, TCC and OCM are performed for patients who require thoracotomy in the setting of severe circulatory failure, the lethal triad, and a requirement of intrapleural hemostasis with gauze packing and prompt intervention for concomitant injuries. In this study, TCC and OCM allowed prompt completion of initial chest surgery and prompt intervention for concomitant injuries; moreover, no empyema or wound infection, which are concerns in TCC and OCM, were observed.

Only a fraction of chest trauma cases reportedly requires emergency surgery for the initial treatment [10–13], and among those that require chest surgery, TCC and OCM are performed in 4–5% of cases, or only 0.01% of all chest trauma cases [15]. Because of this low incidence, the application of DCTS for severe chest trauma has lagged behind that for other types of trauma. However, recent years have seen an increase in reports of DCTS [18], applied in approximately 40% of cases in which the patient requires emergency thoracotomy [19]. However, TCC and OCM have no established indications, and in cases requiring DCTS, their application, including the use of gauze packing, is currently determined at each facility on a case-by-case basis.

The advantages of TCC and OCM include early completion of initial chest surgery for prompt ICU resuscitation, rapid intervention for concomitant injuries, and avoidance of thoracic compartment syndrome due to intrathoracic gauze packing [20–22]. Almost all patients who require DCTS have severe multiple trauma, and a prompt approach to the non-chest trauma site is required. In this study, the initial surgical intervention for the thoracic injury was completed in approximately 1 h via TCC and OCM, and subsequent hemostasis could be promptly initiated via laparotomy and IVR.

Thoracic compartment syndrome, which is very rare in cases of thoracic trauma, is a potentially fatal condition caused by increased airway pressure, decreased ventilation, and decreased cardiac output due to increased intrathoracic pressure [26]. Thus, in theory, thoracic packing may impair cardiac function and increase peak airway pressure, resulting in impaired cardiopulmonary function; however, combining it with TCC and OCM reportedly prevents an increase in the peak inspiratory pressure [22]. Regarding treatment outcomes, the mortality rate among patients undergoing DCTS is 20–60% [15, 16, 18–22], and it is 30–60% when considering only cases to TCC and OCM [18–22]; thus, the 30% mortality rate in this study may be considered reasonable. Another complication related to TCC and OCM management, including gauze packing, is infection of the surgical site. Previous reports have indicated relatively high rates of empyema (0–40% [18–21]) and wound infection (approximately 0–18% [15, 20, 21]). In one study of 12 cases, no infectious complications were observed despite intrapleural gauze packing [21], and in another study, no significant differences in infectious complications were observed between the TCC and OCM group and the definitive thoracotomy group [22]. Factors contributing to the low rate of complications include the short time (52.7 h) until packing gauze was removed and the administration of prophylactic antibiotics in both groups [21, 22]. A multicenter trial indicated that TCC methods involving closure of the skin and muscle layers result in a higher incidence of infectious complications than methods involving skin closure only, adhesive dressing, a negative-pressure wound therapy device, or a commercial device for open abdominal management [18]. The authors of that study suggested that infections complications may be prevented during TCC by avoiding closure of the muscle layer, changing the gauze every 48–72 h, and administering prophylactic antibiotics. None of the patients in our series developed empyema or a wound infection. At our institution, the following factors might have contributed to the avoidance of infectious complications during TCC: the intrapleural gauze was removed and the thoracic cavity, including the wound site, irrigated within 24 h of OCM initiation; the OCM duration was minimized (a median of 2 days); and the muscle layer was not closed. Additionally, although prophylactic antibiotic administration during OCM is not mandatory at our institution, antibiotics were administered in most cases for chest or concomitant injuries.

No consensus has been established for TCC methods, and studies vary widely in terms of whether only the skin is closed and whether an adhesive dressing, a Bogota bag, Barker vacuum-packing, a negative-pressure wound therapy device [17–22], or a commercial device for open abdominal management [27] is used. In our hospital, we use negative-pressure wound therapy devices and a commercial device for open abdominal management because medical devices have not been developed specifically for TCC and OCM. As the use of these devices for TCC and OCM is strictly off-label, we have to follow appropriate measures in accordance with medical ethics, and we will continue to monitor patients closely for the occurrence of complications and for the safety of the treatment.

## Limitations

The most important limitation of this study is its single-center nature and inclusion of only 10 cases. The results of this study suggest that rapid completion of the initial thoracic surgery is beneficial in that it enables prompt intervention for concomitant injuries and initiation of resuscitation treatment in the ICU. However, whether this leads to an improved prognosis or survival is unclear, as is whether TCC and OCM prevent thoracic compartment syndrome. In fact, none of the published reports have indicated that OCM with TCC yielded an improved prognosis, although the mortality rates were lower than those predicted by the Revised Trauma Score and ISS in one study [20]; in another retrospective cohort study, although the number of DCTS cases increased over time, no evidence was reported of improved survival rates [18]. In addition, most of the patients in this study had blunt multiple trauma; however, the previous reports on TCC and OCM included many patients with penetrating injuries; such differences in patient backgrounds should be considered when results are compared between studies. The usefulness of TCC and OCM for cases of severe trauma requires further case accumulation and comparative studies. Outstanding issues include the appropriate indications for OCM and TCC, as well as the standardization of TCC and OCM methods.

## Conclusion

At our institution, we perform OCM with TCC in combination with intrapleural gauze packing for patients who require DCTS for severe trauma. OCM with TCC enables prompt completion of the initial chest surgery and initiation of treatment for concomitant injuries and resuscitation in the ICU. None of the 10 patients exhibited empyema or wound infections.

## Data Availability

The data that support the findings of this study are available from the corresponding author upon reasonable request.

## References

[1] Rotondo MF, Schwab CW, McGonigal MD, Phillips III GR, Fruchterman TM, Kauder DR, et al. ‘Damage control’: an approach for improved survival in exsanguinating penetrating abdominal injury. J Trauma. 1993;35: 375–82 **(discussion 382–3)**. 8371295

[2] Shapiro MB, Jenkins DH, Schwab CW, Rotondo MF. Damage control: collective review. J Trauma. 2000;49:969–78. 11086798 10.1097/00005373-200011000-00033

[3] Roberts DJ, Ball CG, Feliciano DV, Moore EE, Ivatury RR, Lucas CE, et al. History of the innovation of damage control for management of trauma patients: 1902–2016. Ann Surg. 2017;265:1034–44. 27232248 10.1097/SLA.0000000000001803

[4] Benz D, Balogh ZJ. Damage control surgery: current state and future directions. Curr Opin Crit Care. 2017;23:491–7. 29035926 10.1097/MCC.0000000000000465

[5] Rasmussen TE, Clouse WD, Jenkins DH, Peck MA, Eliason JL, Smith DL. The use of temporary vascular shunts as a damage control adjunct in the management of wartime vascular injury. J Trauma. 2006;61:8–12 **(discussion 12–5)**. 10.1097/01.ta.0000220668.84405.17 16832244

[6] Waibel BH, Rotondo MF. Damage control in trauma and abdominal sepsis. Crit Care Med. 2010;38:S421–30. 20724875 10.1097/CCM.0b013e3181ec5cbe

[7] Risinger WB, Smith JW. Damage control surgery in emergency general surgery: what you need to know. J Trauma Acute Care Surg. 2023;95:770–9. 37439768 10.1097/TA.0000000000004112

[8] Scalea TM. Optimal timing of fracture fixation: have we learned anything in the past 20 years? J Trauma. 2008;65:253–60. 18695459 10.1097/TA.0b013e31817fa475

[9] Roberts DJ, Bobrovitz N, Zygun DA, Ball CG, Kirkpatrick AW, Faris PD, et al. Indications for use of thoracic, abdominal, pelvic, and vascular damage control interventions in trauma patients: a content analysis and expert appropriateness rating study. J Trauma Acute Care Surg. 2015;79:568–79. 26402530 10.1097/TA.0000000000000821

[10] Kulshrestha P, Munshi I, Wait R. Profile of chest trauma in a level I trauma center. J Trauma. 2004;57:576–81. 15454805 10.1097/01.ta.0000091107.00699.c7

[11] Demirhan R, Onan B, Oz K, Halezeroglu S. Comprehensive analysis of 4205 patients with chest trauma: a 10-year experience. Interact Cardiovasc Thorac Surg. 2009;9:450–3. 19541693 10.1510/icvts.2009.206599

[12] Bayer J, Lefering R, Reinhardt S, Kühle J, Südkamp NP, Hammer T, et al. Severity-dependent differences in early management of thoracic trauma in severely injured patients—analysis based on the TraumaRegister DGU^®^. Scand J Trauma Resusc Emerg Med. 2017;25:10. 28148274 10.1186/s13049-017-0354-4 PMC5288852

[13] Fokkema AT, Johannesdottir BK, Wendt K, Haaverstad R, Reininga IHF, Geisner T. Comorbidities, injury severity and complications predict mortality in thoracic trauma. Eur J Trauma Emerg Surg. 2023;49:1131–43. 36527498 10.1007/s00068-022-02177-6 PMC10175434

[14] O’Connor JV, DuBose JJ, Scalea TM. Damage-control thoracic surgery: management and outcomes. J Trauma Acute Care Surg. 2014;77:660–5. 25494414 10.1097/TA.0000000000000451

[15] Garcia A, Martinez J, Rodriguez J, Millan M, Valderrama G, Ordoñez C, et al. Damage-control techniques in the management of severe lung trauma. J Trauma Acute Care Surg. 2015;78:45–50 **(discussion 50–1)**. 10.1097/TA.0000000000000482 PMC4279445 25539202

[16] Douglas A 2nd, Puzio T, Murphy P, Menard L, Meagher AD. Damage control thoracotomy: a systematic review of techniques and outcomes. Injury. 2021;52:1123–7. 33386155 10.1016/j.injury.2020.12.020

[17] Ghneim MH, O’Connor JV, Scalea TM. Damage control thoracic surgery: what you need to know. J Trauma Acute Care Surg. 2025;98:11–9. 39375907 10.1097/TA.0000000000004458

[18] Douglas AD, Puzio T, Murphy P, Nahmias J, Bugaev N, Kaafarani H, et al. Damage control thoracotomy trends, techniques, and outcomes: an EAST multicenter trial. J Trauma Acute Care Surg. 2025. 10.1097/TA.0000000000004492. 40435348 10.1097/TA.0000000000004492

[19] Douglas AD 2nd, Puzio TJ, Murphy PB, Kinnaman GB, Meagher AD. Pack the chest: damage control strategy for management in thoracic trauma. Injury. 2024;55:111490. 38523031 10.1016/j.injury.2024.111490

[20] Vargo DJ, Battistella FD. Abbreviated thoracotomy and temporary chest closure: an application of damage control after thoracic trauma. Arch Surg. 2001;136:21–4. 11146769 10.1001/archsurg.136.1.21

[21] Moriwaki Y, Toyoda H, Harunari N, Iwashita M, Kosuge T, Arata S, et al. Gauze packing as damage control for uncontrollable haemorrhage in severe thoracic trauma. Ann R Coll Surg Engl. 2013;95:20–5. 23317720 10.1308/003588413X13511609956057 PMC3964630

[22] Lang JL, Gonzalez RP, Aldy KN, Carroll EA, Eastman AL, White CQ, et al. Does temporary chest wall closure with or without chest packing improve survival for trauma patients in shock after emergent thoracotomy? J Trauma. 2011;70:705–9. 21610362 10.1097/TA.0b013e31820e89f1

[23] Deane M, Galvagno SM Jr, Moran B, Stein DM, Scalea TM, O’Connor JV. Shock, not blood pressure or shock index, determines the need for thoracic damage control following penetrating trauma. Shock. 2020;54:4–8. 31693631 10.1097/SHK.0000000000001472

[24] Manzano-Nunez R, Chica J, Gómez A, Naranjo MP, Chaves H, Muñoz LE, et al. The tenets of intrathoracic packing during damage control thoracic surgery for trauma patients: a systematic review. Eur J Trauma Emerg Surg. 2021;47:423–34. 32594214 10.1007/s00068-020-01428-8

[25] Karmy-Jones R, Namias N, Coimbra R, Moore EE, Schreiber M, McIntyre R Jr., et al. Western Trauma Association critical decisions in trauma: penetrating chest trauma. J Trauma Acute Care Surg. 2014;77:994–1002. 25423543 10.1097/TA.0000000000000426

[26] Wandling MW, An GC. A case report of thoracic compartment syndrome in the setting of penetrating chest trauma and review of the literature. World J Emerg Surg. 2010;5:22. 20673346 10.1186/1749-7922-5-22 PMC2917402

[27] Fernandez LG, Norwood SH, Orsi C, Heck M, Gonzalez K, Williams N, et al. Use of a modified ABTHERA ADVANCE™ open abdomen dressing with intrathoracic negative-pressure therapy for temporary chest closure after damage control thoracotomy. Am J Case Rep. 2022;23:e937207. 36153642 10.12659/AJCR.937207 PMC9520634

